# How Do Empowered Leaders Influence the Job Satisfaction of Kindergarten Teachers in China? Evidence From Mediation Analysis

**DOI:** 10.3389/fpsyg.2020.586943

**Published:** 2021-01-11

**Authors:** Li Liu, Chuan Yang, Dawei Huang

**Affiliations:** ^1^Institute of Education, Yibin University, Yibin, China; ^2^Institute of Education, Sichuan Normal University, Chengdu, China; ^3^Institute of Education, Guizhou Normal University, Guiyang, China; ^4^School of Psychology, Central China Normal University, Wuhan, China

**Keywords:** kindergarten teacher, empowering leadership, job satisfaction, vigor, affective commitment, mediation model

## Abstract

Given the current shortage of kindergarten teachers, this study examines the relationship between empowering leadership and job satisfaction among kindergarten teachers in China from the perspective of their job characteristics and the Chinese cultural context. The participants were 557 Chinese kindergarten female teachers whose average number of years of experience was 2.82 (*SD* = 3.02). They completed a self-report survey regarding empowering leadership, vigor, affective commitment, and job satisfaction. The study results show that vigor and affective commitment could mediate the relationship between empowering leadership and job satisfaction. Finally, we discuss the findings of this study in depth. At the same time, we suggest that administrators should focus on the empowering leadership of Chinese kindergarten teachers, strive to increase their level of dynamism, and finally, strengthen the emotional connection between the organization and the Chinese kindergarten teachers. Thus, we suggest that educational administrators should empower kindergarten teachers more, rather than allowing them to be mere enforcers. In addition, how to enhance the individual dynamics of early childhood teachers and their emotional connection to the organization may also be something for educational administrators to consider.

## Introduction

How to improve teacher job satisfaction has been a topic of concern in many studies. Satisfied teachers are able to accept more job demands (potential stressors) and face less emotional exhaustion (Skaalvik and Skaalvik, 2017a). Furthermore, existing research shows that teachers who are satisfied with their work can generate more positive effects for their students (e.g., student school engagement) (Shoshani and Eldor, 2016). Finally, when the level of job satisfaction is high, teachers are less likely to quit (Skaalvik and Skaalvik, 2017b). The problem today is that many Chinese kindergarten teachers may be severely overworked (Li et al., 2019). A recent government report shows that by the end of 2018, at least 520,000 kindergarten teachers were needed to properly implement kindergarten education (Chen, 2019). In this context, increasing the job satisfaction of kindergarten teachers may be an effective solution to temporarily alleviate the shortage of teachers.

Although many factors have an impact on kindergarten teachers’ job satisfaction, we believe that empowering leadership may be an antecedent that cannot be ignored, given the characteristics of kindergarten teachers’ jobs and the Chinese cultural context. Empowering leadership refers to the sharing of power between the organization or team leaders and their subordinates (Vecchio et al., 2010). First of all, the job of a kindergarten teacher is a process of re-creating knowledge. They need to present book knowledge or skills that children need to learn in a new and understandable way. This process requires strong creativity and motivation on the part of the kindergarten teacher. The redistribution of organizational power that comes with empowering leadership helps to increase work engagement and thus creativity (Gkorezis, 2016), making it easier for employees to feel satisfied with their work (Atik and Celik, 2020; Zhou and Song, 2020). Second, in the Chinese cultural context, high power distance is a distinctive feature (Lin, 2020). In Chinese family life, parental authority over children is widely recognized and children are expected to fulfill their obligations to support their parents; in the workplace, subordinates are expected to follow the arrangements and instructions of their superiors. In a context of high power distance, teachers are more likely to be dissatisfied with their jobs (Wang and Zhang, 2020). A similarly high level of power distance exists in the Chinese kindergarten teacher population (Li and Chen, 2020). Research from organizational psychology confirms that empowering leadership enables employees to feel cared for and supported, narrowing the power distance between superiors and subordinates (Luo and Xiao, 2020). Based on these two points, the primary purpose of this study is to investigate the relationship between empowering leadership and Chinese kindergarten teachers’ job satisfaction.

In addition, focusing only on the direct relationship between empowering leadership and job satisfaction among Chinese kindergarten teachers may be lacking, so we also wanted to explore what specific mechanisms might be at play in their relationship. This will help us to identify some more concrete educational recommendations at the practice level. As mentioned earlier, empowering leadership may lead to increased job satisfaction by increasing kindergarten teachers’ work engagement and innovation. Therefore, we also want to look for a possible mediating factor from this perspective. If there is a common source of both work engagement and innovation, it may be vigor (Shirom, 2011). Vigor is considered to be the sum of the physical strength, emotional energy, and cognitive liveliness that an individual experiences during work (Shirom, 2011). First, an individual’s work engagement requires strong physical energy (Leijten et al., 2015). Second, an individual’s innovative behavior at work requires flexible cognitive abilities (Liu et al., 2020). Finally, the emotional support provided by empowering leadership also contributes to the activation of the individual’s emotional energy (Chow, 2018). In addition, from a cultural context perspective, previous studies have confirmed that high power distance has an inhibitory effect on positive work behaviors (e.g., job performance and voice behaviors) (Bai et al., 2017; Zhou and Liao, 2018). Empowering leadership can reduce the negative effects of power distance (Luo and Xiao, 2020), stimulating individual vigor. On the other hand, the positive relationship between vigor and job satisfaction is also supported by much evidence (Härtel et al., 2009; Isoard-Gautheur et al., 2020). Therefore, vigor may be a mediating variable between empowering leadership and job satisfaction among Chinese kindergarten teachers.

In addition to vigor, there is an intermediate factor that may also be worth noting based on the Chinese cultural context—affective commitment. Affective commitment refers to a psychological state that involves the relationship between individuals and organizations, which is mainly based on emotional attachment (Meyer et al., 1990). In the Chinese cultural context, empowering leadership is often seen as a form of social support (from the leader), but it is also emotionally encouraging (Chow, 2018). Traditional Chinese culture emphasizes the act of repaying kindness (Pan et al., 2016). Social support for the individual in the Chinese cultural context is not just a one-way support, it also implies an obligation to reciprocate (Jing and Chen, 2008). That is to say, the individual who receives social support has an obligation to return the favor to the person who gave it. Many studies of East Asian cultures support this view (Miller and Bersoff, 1992; Chen et al., 2012; Park et al., 2013). As a result, Chinese kindergarten teachers may reciprocate their leaders’ emotional support with affective commitment. According to self-identity theory, when an individual feels a sense of identification with the organization, his job satisfaction is also in a more positive state (Luo et al., 2014; Di et al., 2020). Therefore, the affective commitment of Chinese kindergarten teachers may be another important factor in the relationship between empowering leadership and job satisfaction.

A final issue that requires clarification may be the relationship between vigor and affective commitment. First, according to the previous definition, vigor can serve as an indicator of an individual’s emotional energy in an organizational setting. affective commitment, as an emotional connection between individual and organization, necessarily requires the individual to expend emotional energy to maintain it. Therefore, if an individual’s vigor level decreases, it can be expected that affective commitment may also decrease (Scrima et al., 2014). Second, according to conservation of resources theory, individuals who are working with low levels of vigor are likely to experience high levels of job stress (Shirom, 2011; Hobfoll et al., 2018). Work stress may lead to a decrease in an individual’s level of affective commitment (Yang et al., 2017). Therefore, vigor may increase the affective commitment of Chinese kindergarten teachers.

**FIGURE 1:**
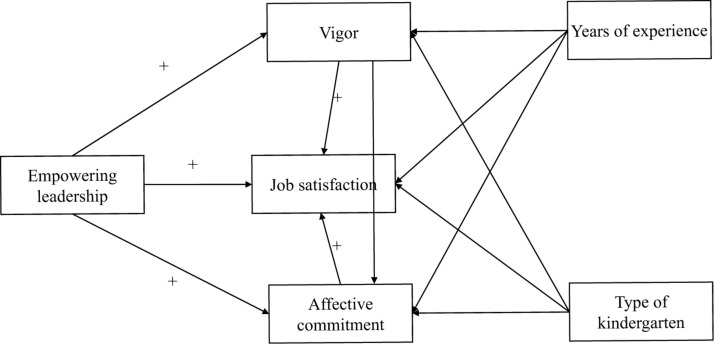
Conceptual model. The model predicts that empowering leadership will be positively related to vigor, job satisfaction, and affective commitment. Vigor will be positively related to job satisfaction and affective commitment will be positively related to job satisfaction. Years of experience and kindergarten type are control variables.

In summary, the current study aimed to explore the relationship between empowering leadership and job satisfaction among kindergarten teachers in China, and the possible mediating roles of vigor and affective commitment. Past studies have shown that a teacher’s years of experience and the type of kindergarten he/she works in (e.g., public or private) can have a significant impact on work attitudes, so years of experience and type of kindergarten were added to the statistical analysis process as control variables (Renzulli et al., 2011; Demirel, 2014). The specific assumptions and conceptual model (Figure 1) of this research were as follows:

• Hypothesis 1: there is a positive relationship between empowering leadership and kindergarten teacher job satisfaction.
• Hypothesis 2: vigor will mediate the relationship between empowering leadership and job satisfaction.
• Hypothesis 3: affective commitment will mediate the relationship between empowering leadership and job satisfaction.
• Hypothesis 4: vigor and affective commitment will mediate the relationship between empowering leadership and job satisfaction.

## Materials and Methods

### Participants and Procedure

In total, 570 teachers were randomly selected from our college’s summer kindergarten teacher training activities. They were informed of the specific situation of the survey by email and asked for their consent. At the end of the survey, each participant was presented a small gift worth 5 yuan. Because of the low proportion of male kindergarten teachers, only female kindergarten teachers, excluding nurses, were selected as the participants for this study. The researchers used face-to-face methods to distribute and collect the questionnaires, and a total of 564 kindergarten teachers volunteered to participate in the study. Finally, we excluded incomplete answers and indiscriminate cases and recovered 557 valid cases. Among them, the average number of years of experience was 2.82 (*SD* = 3.02), 20.3% of the respondents were married (missing = 3.5%), and 27.8% of the respondents were teachers in public kindergartens (the rest were private school teachers). All teachers were the main undertakers of teaching activities.

This research program passed ethical review through the Education Research Ethics Committee in the Faculty of Education at Yibin University. The entire research process was fully compliant with the American Psychological Association’s experimental ethics and the Declaration of Helsinki. Before the investigation, participants were told that they had the right to refuse to participate and to withdraw at any time during the process without any negative consequences (all participants recruited participated in the study, no one dropped out). participants completed an informed consent process.

### Measures

#### Empowering Leadership

This study measured by using the Chinese version of the Empowering Leadership Questionnaire (Zhao and Zhao, 2012). It consists of five self-report items, which are answered using a five-point Likert-type scale (“Strongly disagree” to “Strongly Agree”). Higher scores indicate a higher level of empowering leadership. Some example items were as follows: (1) In my organization, teachers regard leaders as their colleagues; (2) Every teacher in my organization is likely to be a leader; (3) In my organization, teachers share a common responsibility toward the organization; (4) In my organization, every teacher has the opportunity to utilize her abilities; and (5) My organization often makes decisions through group discussions. In this example, the scale has a Cronbach coefficient of 0.745. In addition, the measurement model of the scale also shows a good fit (χ^2^ = 20.506, *df* = 5, *p* < 0.001, *CFI* = 0.978, *TLI* = 0.956, *SRMR* = 0.024, and *RMSEA* = 0.076).

#### Vigor

For this study, we adopted the concept of vigor from the Shirom-Melamed Vigor Measure (Perrewe and Ganster, 2003), which has a reliable and valid Chinese version (Du, 2011). The scale has three dimensions: emotional energy (5-item), cognitive liveliness (3-item), and physical strength (5-item). This measure is answered using a five-point Likert-type scale (“Strongly disagree” to “Strongly Agree”). Higher scores indicate higher levels of vigor in the workplace. In this example, the scale has a Cronbach coefficient of 0.913. In addition, the measurement model of the scale also shows a good fit (χ^2^ = 245.382, *df* = 62, *p* < 0.001, *CFI* = 0.946, *TLI* = 0.932, *SRMR* = 0.036, and *RMSEA* = 0.074).

#### Job Satisfaction

One single-item overall measure was used for assessing job satisfaction (“All in all, I am satisfied with my present job”). Existing research has shown that job satisfaction can be measured reliably with adequate validity by using single-item measures (Dolbier et al., 2005).

#### Affective Commitment

Affective commitment was measured using the Chinese version of the Organizational Commitment Scale (Ling et al., 2001). The affective commitment subscale is a part of the Organizational Commitment Scale. It consists of five self-report items, which are answered by using a five-point Likert-type scale (“Strongly disagree” to “Strongly Agree”). Higher scores indicate higher levels of affective commitment. In this example, the scale has a Cronbach coefficient of 0.786. In addition, the measurement model of the scale also shows a good fit (χ^2^ = 13.243, *df* = 5, *p* = 0.021, *CFI* = 0.977, *TLI* = 0.954, *SRMR* = 0.024, and *RMSEA* = 0.055).

### Data Analysis

For all the variables, descriptive analyses and Pearson’s correlations were carried out using SPSS 22.0. The mediation model was examined using the M*plus*8 with 2,000 bias-corrected bootstrap samples was applied. A *p* value of 0.05 and a 95% confidence interval were set as the critical levels for statistical significance. There are four main indicators (cut-off values) for evaluating structural equation models: comparative fit index (CFI, ≥ 0.9), Tucker–Lewis index (TLI, ≥ 0.9), root mean square error of approximation (RMSEA, ≤ 0.08), standardized root mean-square residual (SRMR, ≥ 0.1). When all indicators meet the cutoff value or better, the model fits better (Wang and Wang, 2020). Given the large number of items on the vigor variable, we used an item packaging strategy. The scores for each indicator were calculated from the sum of the scores of the items of the corresponding dimension.

## Results

Table 1 shows the descriptive statistics with regard to and the correlations between the selected variables. Empowering leadership was positively associated with vigor (*r* = 0.355), job satisfaction (*r* = 0.339), and affective commitment (*r* = 0.527), Therefore, H1 was supported. Vigor was positively associated with job satisfaction (*r* = 0.542) and affective commitment (*r* = 0.536). Job satisfaction was positively associated with affective commitment (*r* = 0.573). The type of kindergarten was negatively associated with all the variables. Years of experience was positively associated with vigor, but this association was weak (*r* = 0.113).

**TABLE 1:** Descriptive statistics and the relationship between variables (*N* = 557).

	1	2	3	4	5	6
1. Vigor	–					
2. Job satisfaction	0.542***	–				
3. Affective commitment	0.536***	0.573***	–			
4. Empowering leadership	0.355***	0.339***	0.527***	–		
5. Type of kindergarten	−0.114*	−0.202***	−0.204***	−0.163***	–	
6. Years of experience	0.113**	0.083	0.144**	0.064	−0.173***	
Mean	50.710	3.784	18.284	18.890	1.721	2.822
Standard deviation	6.663	0.933	3.470	3.261	0.454	3.032

*p < 0.05, **p < 0.01, and ***p < 0.001.

**FIGURE 2:**
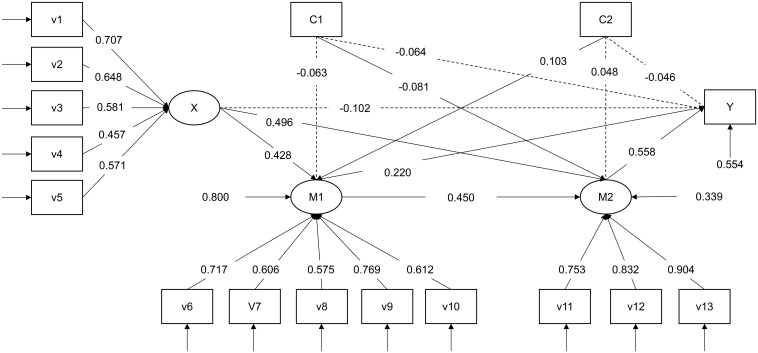
Standardized parameter estimates of the final model. X, empowering leadership; M1, vigor; M2, affective commitment; Y, job satisfaction; C1, type of kindergarten; C2, years of experience. The dashed line represents *p* > 0.05 and the solid line represents *p* < 0.05.

Next, the mediation model was tested in Figure 2. The indices produced by the SEM showed a good fit [χ^2^ = 176.721, *df* = 80, *p* < 0.001; *RMSEA* = 0.047, 90%CI = (0.037, 0.056), *CFI* = 0.957, *TLI* = 0.944, and SRMR = 0.045]. After controlling for the influence of teachers’ years of experience and type of kindergarten, empowering leadership significantly predicted vigor [β = 0.428, *SE* = 0.050, 95%CI = (0.331, 0.525), and *p* < 0.001], and vigor significantly predicted job satisfaction [β = 0.220, *SE* = 0.061, 95%CI = (0.097, 0.342), and *p* < 0.001]; empowering leadership significantly predicted affective commitment [β = 0.496, *SE* = 0.046, 95%CI = (0.405, 0.586), and *p* < 0.001], and affective commitment significantly predicted job satisfaction [β = 0.558, *SE* = 0.086, 95%CI = (0.390, 0.726), and *p* < 0.001], and vigor significantly predicted affective commitment [β = 0.450, *SE* = 0.047, 95%CI = (0.359, 0.542), and *p* < 0.001], However, empowering leadership did not significantly predict job satisfaction [β = −0.102, *SE* = 0.069, 95%CI = (−0.237, 0.033), and *p* = 0.139]. The type of kindergarten significantly predicted affective commitment [β = −0.081, *SE* = 0.037, 95%CI = (−0.154, −0.020), and *p* = 0.029); the years of experience significantly predicted vigor [β = 0.103, *SE* = 0.046, 95%CI = (0.013, 0.193), and *p* = 0.025].

Finally, the analysis of mediating effects shows in Table 2. Vigor will mediate the relationship between empowering leadership and job satisfaction. Affective commitment will mediate the relationship between empowering leadership and job satisfaction. Vigor and affective commitment will mediate the relationship between empowering leadership and job satisfaction. The above results support our previous hypothesis.

**TABLE 2:** Indirect effects.

Model pathway	Point estimate	95%CI	*P*
empowering leadership→vigor→affective commitment→job satisfaction	0.108	(0.063, 0.152)	<0.001
empowering leadership→vigor→job satisfaction	0.094	(0.036, 0.152)	<0.001
empowering leadership→affective commitment→job satisfaction	0.277	(0.172, 0.381)	=0.001

*CI, confidence interval.*

## Discussion

First, the results of this study show that there is a positive relationship between empowering leadership and job satisfaction. This is consistent with the existing non-teacher research (Kim and Beehr, 2018). From the kindergarten teachers’ perspective, this result is consistent with the previous expectations of this study. As we speculated in the introduction, the characteristics of kindergarten teachers’ work require more creativity and a lively work environment. Empowering leadership may give kindergarten teachers more freedom and support in their work and reduce the stresses associated with their cultural background, which helps kindergarten teachers to better use their imagination in their work and make their work more than a simple task. Because the individual’s active participation makes work a unique, private thing, work becomes more meaningful (Bai and Wang, 2020). As a result, kindergarten teachers’ job satisfaction increased. Then, to further examine the specific mechanisms by which empowering leadership works, this study included the variable of vigor. Because, if empowering leadership does increase autonomy at work and reduces the oppressive atmosphere brought about by power distance, kindergarten teachers may be more proactive, relaxed, and comfortable in their work attitudes. Obviously, they would be more vigorous. The results of the mediation analysis support this idea that empowering leadership through vigor has a significant impact on job satisfaction. In summary, the mediation effect of vigor reveals, to some extent, the importance of empowering leadership for Chinese kindergarten teachers, which is determined by the nature of their work and the cultural context. Vigor implies a more positive physical state, more active cognitive abilities, and a healthier emotional experience (Shirom, 2011), and empowering leadership may make all of this a reality. However, current research focuses on how to improve job satisfaction among Chinese kindergarten teachers in order to reduce the negative effects of teacher shortages. Perhaps, once China has enough kindergarten teachers, leadership empowerment should also be a factor that cannot be ignored. Thousands of young children need teachers who are not just satisfied with their jobs, but who are energetic, creative, and emotionally positive participants in their development.

Second, this study first hypothesized that the leadership support implied by empowering leadership might increase Chinese kindergarten teachers’ affective commitment to the organization from the perspective of cultural differences in social support. Then, based on self-identity theory, it is hypothesized that emotional commitment to the organization will help Chinese kindergarten teachers feel more satisfied with their jobs (Luo et al., 2014; Di et al., 2020). In addition, considering that affective commitment is a resource-involving behavior, the immediate prerequisite for this behavior may be whether or not kindergarten teachers have sufficient psychological resources. Therefore, vigor, as an external manifestation of a teacher’s psychological resources, should be positively associated with affective commitment. Therefore, we examined the serial mediating role of vigor and affective commitment in the relationship between empowering leadership and job satisfaction among Chinese kindergarten teachers. The results support the mediating role of affective commitment as well as vigor and its serial mediating role. In summary, the mediating role of affective commitment may provide further insight into the possible positive consequences of empowering leadership in the Chinese cultural context. Since ancient times, Chinese culture has emphasized that a true man can dies for one who appreciates him. It is a matter of pride for ordinary people to be valued by their leaders or superiors. This is because it means that the individual is important to the leader or has an important social status in the organization. In order to maintain this reality, the individual tends to work harder to prove his or her worth. In the process, the organization or workplace becomes part of the individual’s self-identity and he naturally develops an emotional identity with the organization. Moreover, the serial mediation of vigor and affective commitment may be due to the sense of support that comes with empowering leadership. This also reveals, to some extent, the common factor behind vigor and affective commitment—psychological resources (Tourigny et al., 2013; Rodríguez-Muñoz et al., 2015). Therefore, in addition to empowering leadership, an effective way to increase the job satisfaction of Chinese kindergarten teachers may be to increase their social support. Because social support is a powerful external psychological resource, it can help individuals cope with stress at work and help them to have a vigorous work state.

Finally, although the direct effect of empowering leadership and job satisfaction among kindergarten teachers in China was not significant in the serial mediation model. For two reasons, we think it is still important to discuss this phenomenon. First, the results of significance tests are not direct estimates of the true (Dienes and Mclatchie, 2018). Thus, a researcher cannot determine that a phenomenon is impossible simply by virtue of a non-significant result. Second, the value of the direct effect is a negative value. Although, empowering leadership initially appeared to researchers as a positive attitude (Spreitzer, 1995; Fineman, 2006), many studies have also confirmed the positive effect of leadership empowerment on individual job satisfaction (Amundsen and Martinsen, 2015; Dahinten et al., 2016). However, our results (a study with Chinese kindergarten) support the claim that there is a negative effect of leadership empowerment (Cheong et al., 2016, 2019). The following is a discussion of the direct effects of this study. In the mediation model, the effect values of empowering leadership and job satisfaction are established under a condition that excludes the effects of vigor and affective commitment. Previous research has found that empowering leadership may cause individuals to feel stressed (Cheong et al., 2019). Here, we attempt to explain this using Eysenck’s (1963) arousal theory, which suggests that extraverted individuals have higher arousal levels and that in order to maintain arousal levels, individuals need to take the initiative to engage in social interactions and achieve psychological balance. Similarly, the level of initiative is not consistent across individuals. Individuals with high levels of initiative need to be more involved and autonomous in their work. As a result, they need more empowering leadership. Individuals with low levels of initiative do not want to show much positive action at work, and may expect a quiet job, and that’s it. Shared leadership authority may disrupt this peacefulness and burden them with a heavy burden. Moreover, in East Asian cultures, the individual’s emphasis on leadership may add to this burden (Cheong et al., 2016). Thus, our study may, to a small extent, account for the fact that empowering leadership is not entirely positive, especially when individuals’ sources of job satisfaction do not include vigor and affective commitment.

## Limitations and Future Research

The study focuses on the relationship between the empowering leadership of kindergarten teachers and their job satisfaction and makes some relevant recommendations. However, this research does have some limitations and deficiencies. First, this study used cross-sectional data. It is necessary to use longitudinal data in order to further confirm the relationship between empowering leadership, vigor, affective commitment, and job satisfaction in the context of kindergarten teachers. Second, in a strict sense, affective commitment includes the emotional identity of kindergarten teachers within their organizations (Allen and Meyer, 1990), but it is still an individual-level variable. This means that the inference of this study does not consider the impact at the organizational level; thus, for now, it should be utilized cautiously in the actual management of kindergarten teachers. In the foreseeable future, some organizational factors will have to be considered in order to improve the job satisfaction of kindergarten teachers in a more systematic way; the factors of organizational level are also advocated by the theory of conservation of resources (Hobfoll et al., 2018). Last, all the study participants are from one Province; this may affect the external validity of the research findings.

## Educational Suggestions

First of all, management should pay attention to the empowering leadership of Chinese kindergarten teachers by giving them more autonomy and support in their work and by giving them space to express themselves in their work. Secondly, improving the vigor status of Chinese kindergarten teachers will help to exert the positive effects of empowering leadership on the one hand, and increase their job satisfaction on the other. Considering the definition of vigor, the administration should take more effective measures in the areas of physical training, mental development, and emotional management to help Chinese kindergarten teachers improve their vigor levels. Finally, although affective commitment is an individual’s emotional identification with an organization, the emotional connection is a two-way street. Management should understand the emotions of kindergarten teachers and provide more support and encouragement to them in their work.

## Data Availability Statement

The raw data supporting the conclusions of this article will be made available by the authors, without undue reservation.

## Ethics Statement

This research program passed ethical review through the Education Research Ethics Committee in the Faculty of Education at Yibin University. The entire research process was fully compliant with the American Psychological Association’s experimental ethics and the Declaration of Helsinki. Before the investigation, participants were told that they had the right to refuse to participate and to withdraw at any time during the process without any negative consequences (all participants recruited participated in the study, no one dropped out). participants completed an informed consent process.

## Author Contributions

LL and CY contributed equally to this work, including research design, and manuscript writing. DH conducted the statistical analysis. All authors contributed to the article and approved the submitted version.

## Conflict of Interest

The authors declare that the research was conducted in the absence of any commercial or financial relationships that could be construed as a potential conflict of interest.
